# A Holistic Systems Approach to Characterize the Impact of Pre- and Post-natal Oxycodone Exposure on Neurodevelopment and Behavior

**DOI:** 10.3389/fcell.2020.619199

**Published:** 2021-01-07

**Authors:** Katherine E. Odegaard, Victoria L. Schaal, Alexander R. Clark, Sneh Koul, Jagadesan Sankarasubramanian, Zhiqiu Xia, Melissa Mellon, Mariano Uberti, Yutong Liu, Andrew Stothert, Matthew Van Hook, Hanjun Wang, Chittibabu Guda, Steven J. Lisco, Gurudutt Pendyala, Sowmya V. Yelamanchili

**Affiliations:** ^1^Department of Anesthesiology, University of Nebraska Medical Center, Omaha, NE, United States; ^2^Department of Genetics, Cell Biology & Anatomy, University of Nebraska Medical Center, Omaha, NE, United States; ^3^Department of Radiology, University of Nebraska Medical Center, Omaha, NE, United States; ^4^Department of Ophthalmology & Visual Sciences, Truhlsen Eye Institute, University of Nebraska Medical Center, Omaha, NE, United States

**Keywords:** oxycodone, electrophysiology, RNA-sequencing (RNA-Seq), Von Frey, proton magnetic resonance spectroscopy (1H-MRS)

## Abstract

**Background:** Increased risk of oxycodone (oxy) dependency during pregnancy has been associated with altered behaviors and cognitive deficits in exposed offspring. However, a significant knowledge gap remains regarding the effect of *in utero* and postnatal exposure on neurodevelopment and subsequent behavioral outcomes.

**Methods:** Using a preclinical rodent model that mimics oxy exposure *in utero* (IUO) and postnatally (PNO), we employed an integrative holistic systems biology approach encompassing proton magnetic resonance spectroscopy (^1^H-MRS), electrophysiology, RNA-sequencing, and Von Frey pain testing to elucidate molecular and behavioral changes in the exposed offspring during early neurodevelopment as well as adulthood.

**Results:** ^1^H-MRS studies revealed significant changes in key brain metabolites in the exposed offspring that were corroborated with changes in synaptic currents. Transcriptomic analysis employing RNA-sequencing identified alterations in the expression of pivotal genes associated with synaptic transmission, neurodevelopment, mood disorders, and addiction in the treatment groups. Furthermore, Von Frey analysis revealed lower pain thresholds in both exposed groups.

**Conclusions:** Given the increased use of opiates, understanding the persistent developmental effects of these drugs on children will delineate potential risks associated with opiate use beyond the direct effects in pregnant women.

## Introduction

Over the last few years, the increasing trend in opioid abuse has become a major public health crisis across the globe. As a result, this steep increase in abuse of prescription opioids, which include both licit and illicit opioids, has resulted in the opioid epidemic (Volkow and McLellan, 2016). Whilst this epidemic has traversed different groups in the society, pregnant women are a particularly vulnerable group since they are prescribed opioids such as morphine, buprenorphine, and methadone, all of which have been shown to cross the placenta (Gerdin et al., 1990; Nanovskaya et al., 2002, 2008), potentially impacting the developing fetus. Limited data exist regarding the effects of *in utero* (IUO) or postnatal (PNO) exposure to oxycodone (oxy), however. Oxy is prescribed for multiple types of pain and can bind to mu- and kappa-opioid receptors (Kim, 2017). Oxy easily passes through the blood-brain barrier, thus allowing higher concentrations to accumulate in the brain (Okura et al., 2008, 2015; Chaves et al., 2017), subsequently contributing to its analgesic properties and risk for dependency and addiction.

Several studies (Byrnes and Vassoler, 2018) have been conducted with rodent models to investigate the detrimental effects of gestational opioid use on neurodevelopment of the offspring, but a gap in knowledge exists regarding the effects of IUO or PNO oxy exposure on synaptogenesis. We have previously identified novel miRNA signatures related to neurodevelopment contained within brain-derived extracellular vesicles of PNO and IUO offspring (Shahjin et al., 2019), and our current study aims to investigate metabolic, synaptic, molecular, and behavioral alterations in these exposed offspring. Using a Sprague Dawley rat model previously established by our labs (Shahjin et al., 2019; Odegaard et al., 2020), we employed proton magnetic resonance spectroscopy (^1^H-MRS) to measure biochemical changes of main brain metabolites in the hippocampus. Additionally, we identified synaptic alterations in the hippocampus through the use of electrophysiology experiments. Further, RNA-sequencing (RNA-seq) was conducted on tissue RNA isolated from the prefrontal cortex (PFC) to determine changes in gene expression, particularly in genes related to neurodevelopment, disease states, and mood disorders. The hippocampus and the PFC are key regions involved in substance abuse disorders and the negative emotional state associated with withdrawal (Koob, 2020); indeed, systemic opioid exposure has been shown to attenuate hippocampal afferent-driven activity in the PFC (Giacchino and Henriksen, 1998). For these reasons, we have investigated both regions in this study to identify alterations in either area of the brain during the early developmental period spanning from post-natal day 14 (P14) to P17, which corresponds with peak synaptogenesis (Semple et al., 2013). In our final experiments, we employed Von Frey tests to elucidate any lasting impacts of early-life oxy exposure on pain thresholds. The comprehensive and systematic approach used in this study allows for thorough research into pre- and post-natal oxy abuse, a critical step in closing the knowledge gap surrounding this commonly used opioid analgesic.

## Methods

### Animals

Male and female Sprague Dawley rats were obtained from Charles River Laboratories Inc. (Wilmington, MA, USA) and group housed in a 12 h light–dark cycle and fed *ad libitum*. The total number of animals used for this study can be found in Supplementary Table 1. All procedures and protocols were approved by the Institutional Animal Care and Use Committee of the University of Nebraska Medical Center (UNMC) and conducted in accordance with the National Institutes of Health Guide for the Care and Use of Laboratory Animals.

### Oxycodone Treatment

The development of the IUO treatment paradigm was adapted from a previously published study (Davis et al., 2010), and the overall treatment paradigm previously established in our lab was followed (Shahjin et al., 2019; Odegaard et al., 2020). Briefly, nulliparous female (64–70 days of age) Sprague Dawley rats were treated with oxycodone HCl (Sigma Aldrich, St. Louis, MO) dissolved in saline or saline vehicle via oral gavage. An ascending dosing procedure was used wherein doses of 10 mg/kg/day of oxy were orally-gavaged for 5 days followed by a 0.5 mg/kg/day escalation for 10 days until reaching a final dose of 15 mg/kg/day, after which females were mated with proven male breeders. The treatment regimen continued throughout mating, gestation, and parturition until weaning (P21). For the PNO paradigm, dams were orally-gavaged with 15 mg/kg/day of oxy only after parturition until weaning. Upon weaning of the pups, dams were euthanized by isoflurane overdose followed by decapitation using a guillotine.

### MRI/MRS Acquisitions

P17 pups were used for *in vivo* localized ^1^H-MRS imaging of the hippocampus. Animals were anesthetized by inhalation of 1–1.5% isoflurane in 100% oxygen and maintained 40–80 breaths/minute. The duration of a study for a single animal was about 1 h. MRI and ^1^H-MRS data were obtained using a Bruker® Biospin 7 Tesla/21 cm small animal scanner (Bruker, Billerica, MA), operating at 300.41 MHz, using a laboratory-built 22 mm diameter quadrature birdcage volume coil. All first- and second-order shim terms were first automatically adjusted in the volume-of-interest (VOI) using MAPSHIM® (Bruker, Billerica, MA), with a final shim performed manually to achieve a water line width of 10–15 Hz. The water signal was suppressed by variable power radiofrequency pulses with optimized relaxation delays (VAPOR) (Tkác et al., 1999). MR images were acquired for anatomical reference using a multi-slice rapid acquisition with relaxation enhancement (RARE) sequence (Effective echo time (TE) = 36 ms, Rare Factor = 8, repetition time (TR) = 4,200 ms, Number of Averages (NA) = 2, Scan Time = 3 m 21 s; FOV = 20 × 20 mm^2^, Matrix Size = 256 × 256, Spatial Resolution = 0.078125 × 0.078125 mm^2^, Number of Slices = 29, Slice Thickness = 0.5 mm). ^1^H MRS data sets were obtained using semiLASER localization with timing parameters (TE/TR = 40/4,000 ms, 576 averages, 2,048 points) from a 2 × 5.187 × 1.557 mm^3^ (16.15 μl) VOI located in the hippocampus. Pulse types and specifications: Excitation: hermite 90, duration = 0.7 ms, bandwidth = 5,400 Hz; 1st and 2nd Refocusing: hyperbolic secant, duration = 4 ms, bandwidth = 9484.5 Hz. The acquisition time was 38:24 min per data set. All pulses were applied with a frequency offset of −600 Hz to center the pulse bandwidth between Creatine (CRE) and N-Acetyl Aspartate (NAA). For the water suppression module, the spoiler strength matrix was calculated automatically. Spoiler strength was 35%; spoiler duration was 1.5 ms. For each experiment, one data set was acquired without water suppression to be used as the water concentration reference for the quantitation process. Unsuppressed water spectra were obtained with identical metabolite spectra parameters except for the following: TR = 10,000 ms, NA = 1, and Receiver Gain = 64. One 64 average (for quality assessment) plus four 128-average data sets were acquired for metabolite measurements using a combination of VAPOR (Tkác et al., 1999) scheme for water suppression.

Model parameters and constraints for quantification were generated using spectra from phantoms (*n* = 14) for the following metabolites: Alanine (ALA), Aspartate (ASP), Gamma-Aminobutyric acid (GABA), Glucose (GLC), Glutamine (GLN), Glutamate (GLU), Glycine (GLY), Lactate (LAC), Myo-inositol (MYO), Phosphorylcholine (PC), Taurine (TAU), total choline (tCHO), CRE, and NAA. Phantoms of each metabolite were prepared in pH 7.5 phosphate buffer (100 mM) and contained *3-(trimethylsilyl)-1-propane-sulfonic acid* and *sodium formate* as chemical shift and phasing references. Spectra for each metabolite at known concentrations were acquired using semiLASER (Wijnen et al., 2010) sequences at 40 ms TE, maintaining the phantom at 38°C with a circulating water jacket during spectral acquisition. The set of metabolite spectra formed a *metabolite basis set*, which was used as *prior-knowledge* in the quantification process. In all groups, *n* = 6 for all metabolites except LAC (IUO *n* = 4).

### Electrophysiology

Coronal hippocampal brain slices were prepared from P17 animals (*n* = 6 per group) using the “protected recovery” method (Ting et al., 2014). Briefly, rats were euthanized by CO_2_ asphyxiation and decapitated; brains were rapidly dissected into a slush of artificial cerebrospinal fluid (ACSF) containing (in mM) 124 NaCl, 2.5 KCl, 1.25 NaH_2_PO_4_, 24 NaHCO_3_, 12.5 glucose, 2 CaCl_2_, and 2 MgSO_4_ and continuously bubbled with a mixture of 5% CO_2_ and 95% O_2_. The cerebellum was removed with a razor blade, and the brain was affixed to the cutting chamber using cyanoacrylate glue. Two hundred and fifty micron-thick coronal brain sections through the hippocampus were cut using a vibrating microtome (Leica VT1000S) and hemisected into right and left halves through the midline before being transferred to a net submerged in an N-methyl-D-glucamine (NMDG)-based ACSF composed of (in mM) 92 NMDG, 2.5 KCl, 1.25 NaH_2_PO_4_ 30 NaHCO_3_, 20 HEPES, 0.5 CaCl_2_, 10 MgSO_4_, 2 thiourea, 5 L-ascorbic acid, and 3 Na-pyruvate, warmed to ~30°C and bubbled with 5% CO_2_ and 95% O_2_. After a 10 to 15 min incubation in the NMDG ACSF, slices were transferred to a chamber containing room temperature ACSF and allowed to recover for 1 h before beginning patch clamp experiments. Reagents were purchased from Thermo Fisher Scientific (Waltham, MA) unless noted otherwise.

For whole-cell recording, slices were positioned in a recording chamber on an upright fixed-stage microscope (Olympus BX51WI) and superfused by a gravity-fed system with ACSF warmed to 29–31°C using an in-line solution heater at approximately 4 mL/min. The ACSF was supplemented with 60 μM picrotoxin. A concentric bipolar stimulating electrode was positioned in the *stratum radiatum* to stimulate Schaffer collateral axons using a 0.1 ms current delivered at 0.1 Hz from an isolated pulse stimulator (A-M Systems Model 2100). CA1 pyramidal neurons were targeted for whole-cell recording with patch pipettes pulled from thin-walled borosolicate glass on a Sutter P-1000 micropipette puller. The patch pipettes had a resistance of 5–8 MΩ when filled with a solution containing (in mM) 120 Cs-methanesolfonate, 10 HEPES, 8 TEA-Cl, 5 ATP-Mg, 0.5 GTP-Na_2_, 5 phosphocreatine, and 0.5 EGTA (pH = 7.35, osmolality = 282 mOsm). Reported voltages are corrected for a 10 mV liquid junction potential. The intensity-response profile of the evoked excitatory post-synaptic currents (EPSCs) for each cell was determined by the average of 3–10 responses obtained at each stimulus strength (50–225 μA). The AMPA/NMDA ratio was measured as the ratio of the peak of the inward EPSC recorded at −70 mV to the outward EPSC amplitude at 50 ms post-stimulus at a holding potential of +40 mV. Miniature EPSCs (mEPSCs) were recorded in the absence of stimulus and were detected and analyzed using MiniAnalysis (Synaptosoft). mEPSC frequency for each recorded cell was determined as the median of the instantaneous frequencies of all detected events for that cell. Due to variability in the number of cells patched and recorded, respective samples sizes are provided in **Figure 2**.

### Total RNA Extraction, Quality Control, Library Preparation, and RNA-Seq

Total RNA from prefrontal cortex (PFC) tissue was isolated from the randomly selected pups (*n* = 6) from each treatment group at P14 using the Direct-Zol RNA kit (Zymo Research, CA, USA) based on the manufacturer's protocol. Samples were sent to UNMC's Next Generation Sequencing (NGS) core. RNAseq libraries were generated beginning with 1 ug of total RNA from each sample using the TruSeq V2 RNA sequencing library kit from Illumina following recommended procedures (Illumina Inc., San Diego, CA). Resultant libraries were assessed for size of insert by analysis of an aliquot of each library on a BioAnalyzer instrument (Agilent Technologies, Santa Clara, CA). Each library contained a unique indexing identifier barcode allowing the individual libraries to be multiplexed together for efficient sequencing. Multiplexed libraries (18 samples per pool) were sequenced on a single flow cell of the NextSeq550 DNA Analyzer (Illumina) to generate a total of ~28 million 75 bp single reads for each sample.

### Bioinformatics

Differentially expressed genes (up- and downregulated) between SAL and PNO, SAL and IUO, and PNO and IUO were chosen for further functional characterization using ClueGO plug-in module (Bindea et al., 2009) in Cytoscape software (Shannon et al., 2003). The “biological process” option in Clue-Go analysis was used to visualize the categories of DEG functions in each comparison.

### Von Frey

Von Frey experiments were conducted (*n* = 4) from each group at P17, and the same animals were tested at P75. The test was commenced as the rats placed four paws comfortably on the mesh floor and the plantar were clearly visible. The examiner randomly picked the left or right hind paw as the first evaluated paw during each assessment. A monofilament Von Frey hair was applied exactly vertical on the plantar surface until the hair buckled and the shape of the hair was held for 5 s. The specific value of the forces chosen were: 0.6, 1.0, 1.4, 2.0, 4.0, 6.0, 8.0, 10.0, and 15.0 g. The cut-off force was set as 15.0 g because the paw would be lifted if the next force (26.0 g) was applied. During the measuring process, each force was applied 10 times with an interval of at least 5 s to allow the animal to recover from the previous stimuli. Noxious responses were determined if any of the following robust reflex responses occurred: paw retracting, paw withdrawal, or paw licking. Once there were 4 positive responses in 10 applications, the force was determined as the mechanical withdrawal threshold. The mechanical withdrawal threshold for both of two paws was recorded.

### Statistical Analyses

All data represented in the manuscript are reported as mean ± SEM. Data in each analysis were normally distributed. Significant differences were computed using Welch's *t*-test (Electrophysiology) and two-way ANOVA (Von Frey and MRI/MRS data) followed by Tukey's test with a significance criterion of *p* ≤ 0.05. All statistical tests were performed with GraphPad Prism (La Jolla, CA, USA); data represented as Mean ± SEM on the graphs.

## Results

### Quantitation of Metabolites in Oxy-Exposed Pups

**Figure 1:**
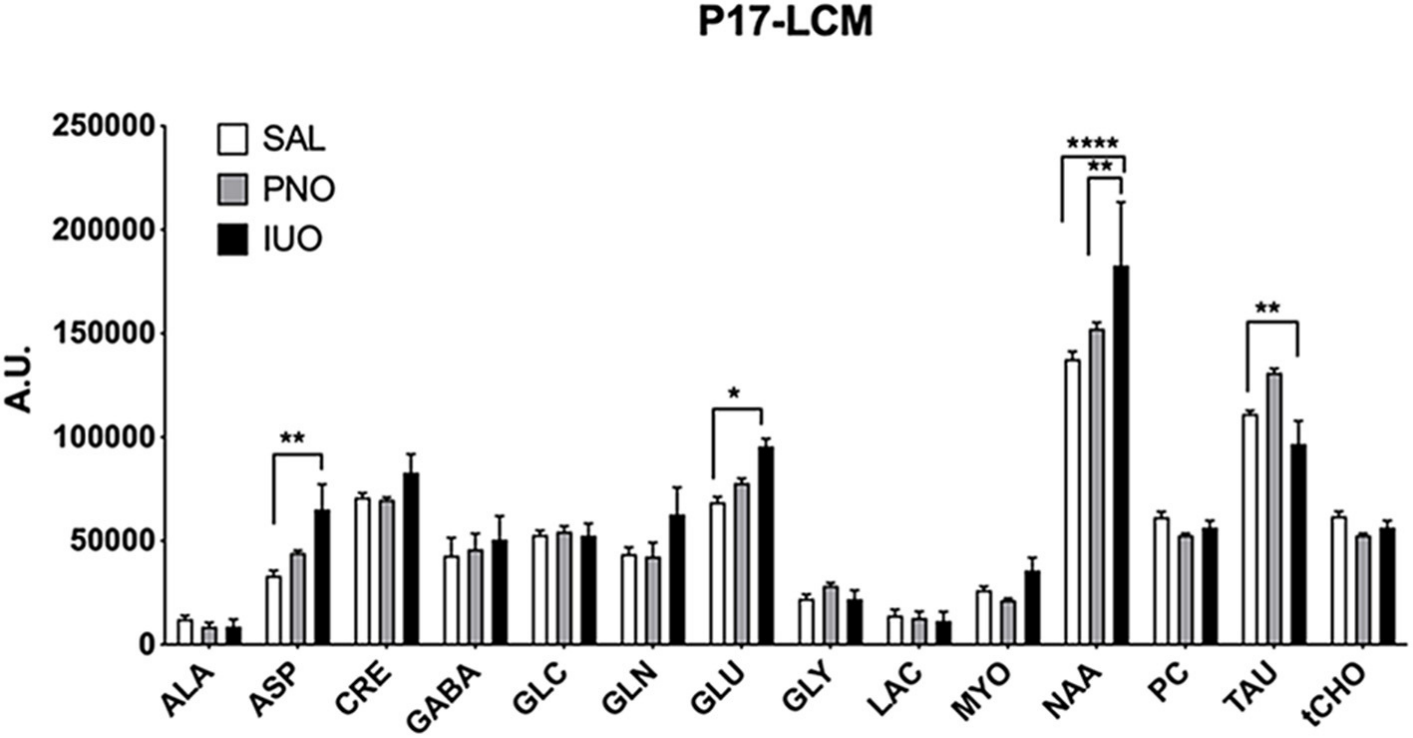
Metabolite concentrations in the hippocampus measured by ^1^H-MRS at P17 in PNO and IUO animals. Measured metabolites included Alanine (ALA), Aspartate (ASP), Creatine (CRE), Gamma-Aminobutyric acid (GABA), Glucose (GLC), Glutamine (GLN), Glutamate (GLU), Glycine (GLY), Lactate (LAC), Myo-inositol (MYO), N-Acetyl Aspartate (NAA), Phosphorylcholine (PC), Taurine (TAU), and total choline (tCHO). In all groups, *n* = 6 for all metabolites except Lac (*n* = 4 in the IUO group). All data represented as mean ± SEM. *p < 0.05, **p < 0.01, ****p < 0.0001.

Brain metabolites are spatiotemporally regulated during development (Miyazawa and Aulehla, 2018). However, it is unknown whether IUO or PNO exposure influences the expression levels of these metabolites in the offspring. Accordingly, we conducted ^1^H-MRS scans on the brain hippocampus of postnatal day 17 (P17) saline, PNO, and IUO groups (Figure 1). We found that IUO or PNO treatment did affect metabolite concentrations in these animals [Metabolite: *F*_(13, 208)_ = 96.75, *P* < 0.0001; Treatment: *F*_(2, 208)_ = 5.520, *P* = 0.0046; Interaction: *F*_(26, 208)_ = 2.093, *P* = 0.0023]. Specifically, we identified higher levels of neurotransmitter aspartate (ASP) and glutamate (GLU) in both PNO and IUO groups. However, N-acetyl aspartate (NAA), the second most abundant metabolite in the brain, was significantly elevated in the IUO group. Additionally, taurine (TAU) concentration was elevated in the PNO group but was significantly lower in the IUO offspring compared to controls. Together, these data point to alterations in key metabolite levels in both the PNO and IUO groups that are more pronounced in the latter.

### Synaptic Alterations in IUO and PNO Offspring

**Figure 2:**
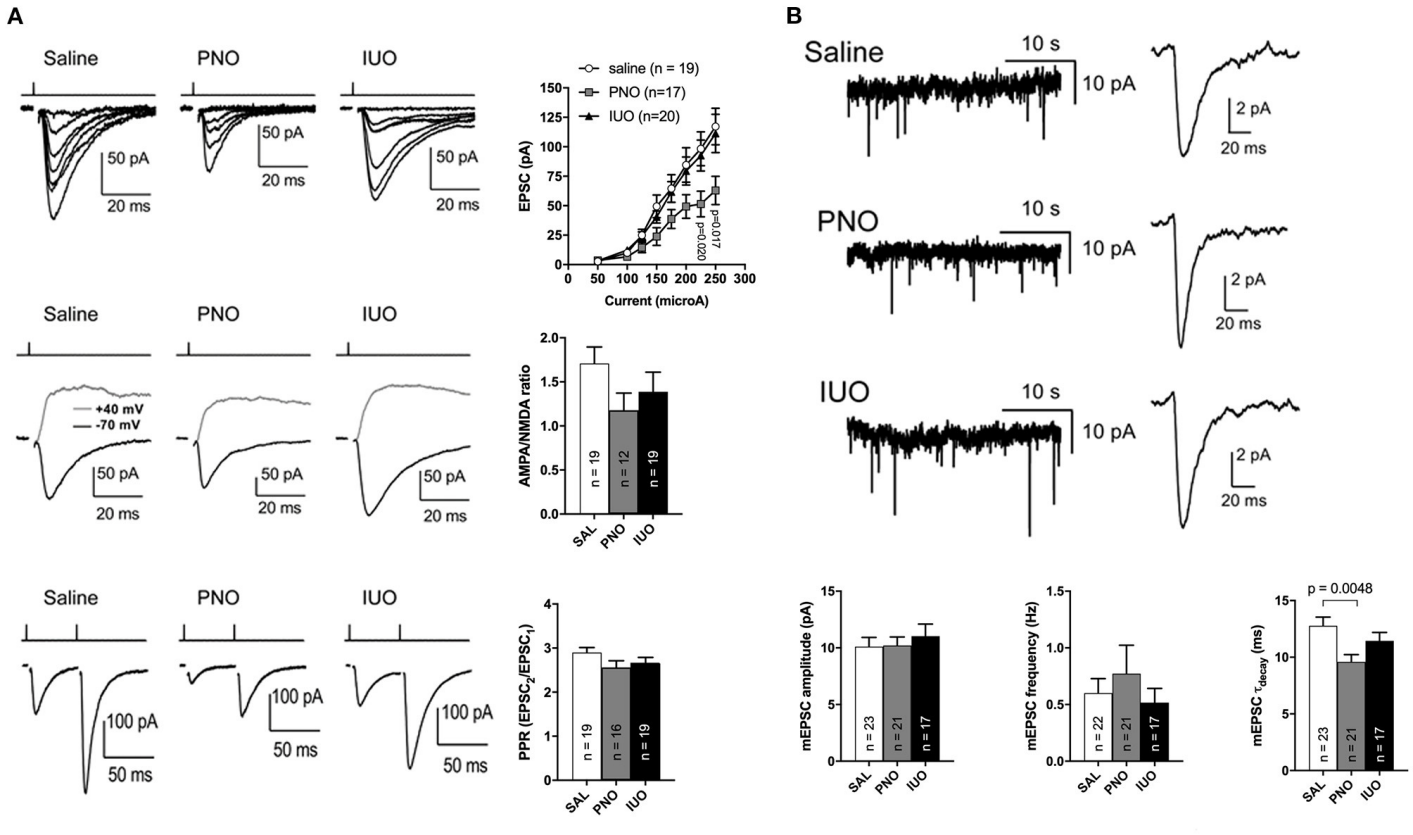
Evoked EPSC and mEPSC monitored in hippocampal slices of control, PNO, and IUO rats. **(A)** Evoked EPSC data; Top: A series of EPSCs recorded in each of the three treatment conditions in response to a series of stimulus strengths (50–225 μA). Stimulus timing is marked above the traces. Group data show the intensity-response profiles for cells recorded from Saline, PNO, and IUO rats. Middle: AMPA receptor and NMDA receptor-mediated EPSCs recorded by voltage-clamping CA1 pyramidal cells at −70 and +40 mV, respectively. The AMPA receptor component was measured at the peak of the inward EPSC while the NMDA receptor component was measured 50 ms post-stimulus. Group data of AMPA/NMDA ratios show there was no significant difference between the groups. Bottom: Paired pulse traces showing the characteristic synaptic facilitation in response to a pair of pulses separated by 50 ms. Group data show the paired pulse ratio was not significantly different between treatment conditions. **(B)** Traces of mEPSCs recorded in the absence of stimulation followed by mEPSCs waveforms from individual cells. These were obtained by averaging all detected events in individual cells. Group data show that mEPSC amplitudes and frequencies were not significantly different between treatment groups. Group data of mEPSC decay time constants (τ_decay_), show that mEPSCs in the PNO group decayed more quickly than in control. Sample sizes of recorded cells are shown in the bars of each graph; all data represented as mean ± SEM.

CA1 synapses were monitored in hippocampal slices of control, PNO, and IUO rats (Figure 2). Input-response curves showed that the post-synaptic currents in the PNO group cells were smaller than control (*p* = 0.02, *p* = 0.017; Figure 2A). Although the AMPA/NMDA ratio in PNO rats appeared reduced compared to controls, the difference was not significant (*p* = 0.07; Figure 2A). The paired pulse ratio (PPR) of post-synaptic currents did not significantly differ among the three groups, suggesting no change in presynaptic vesicle release probability (Figure 2A). When measuring miniature excitatory post-synaptic currents (mEPSCs), we found that, although the frequency and amplitude of the currents did not differ between groups, the PNO mEPSCs had slightly faster decay kinetics (*p* = 0.0048; Figure 2B), which is consistent with altered AMPA receptor subunit composition. Together, these data point to altered synaptic maturation in the PNO offspring.

### RNA-Seq Highlights Gene Expression Changes in Oxy-Exposed Pups

**Figure 3:**
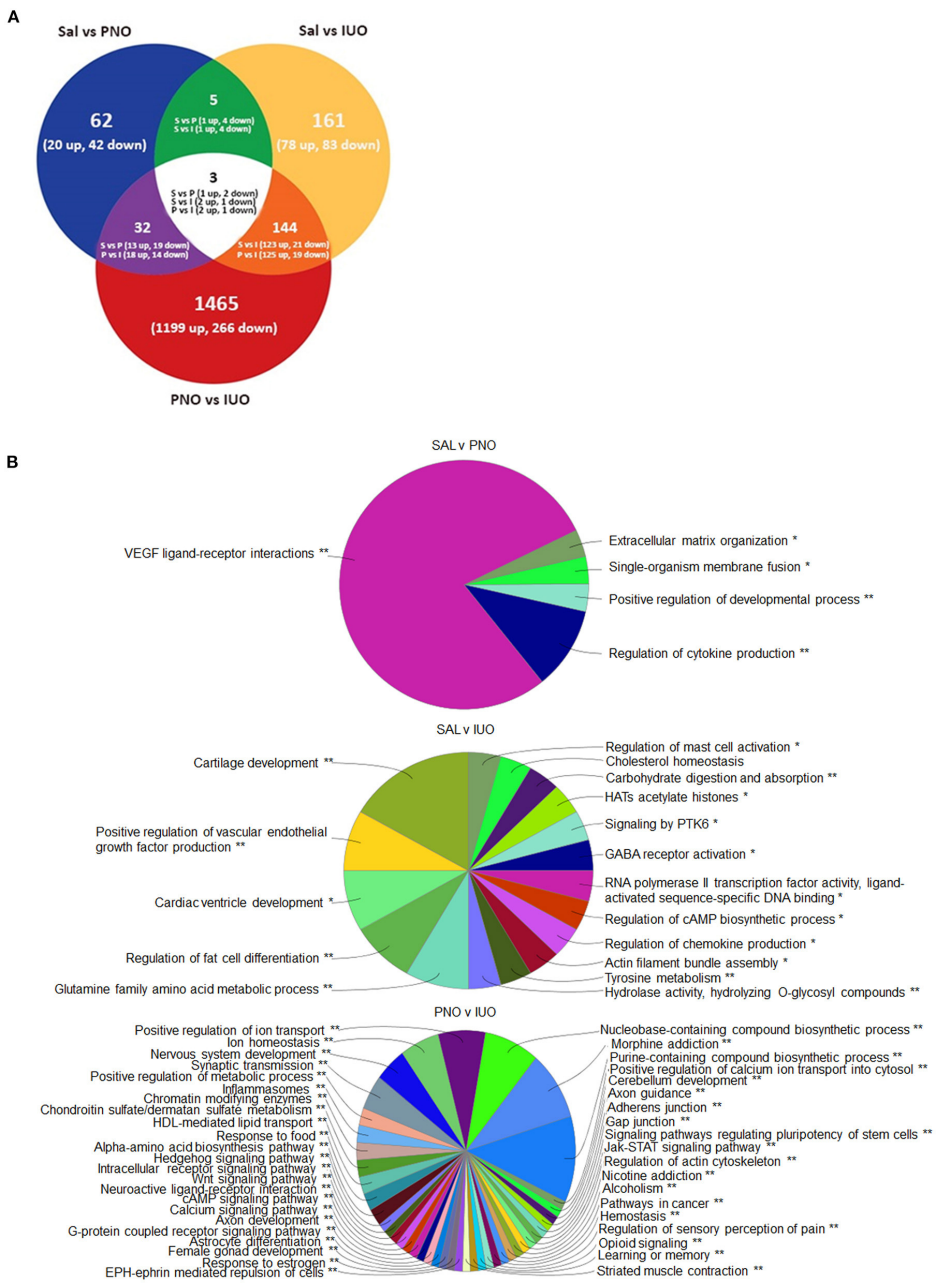
Alterations in gene expression revealed by RNA-seq. **(A)** Differentially expressed genes at P14 identified using RNA-Seq. RNA was isolated from the PFC regions of P14 brains (n = 6) for each group. **(B)** Mapping of biological processes in the PFC. Clue-Go pie diagrams resulting from RNA-seq data show enriched biological processes involved in developmental, neurological, and psychological disorders, which are more impacted in the IUO offspring. The asterisks represent the group term *p*-value representing each category. *p < 0.05, **p < 0.01.

To further understand the molecular causes associated with changes in synaptic currents, we performed RNA-seq analysis on the prefrontal cortex (PFC) (Figure 3A; Supplementary Table 2). Employing a criteria of 1.5-fold change and *p* < 0.05, we found 62 genes (20 up and 42 down) between saline and PNO and 161 genes (78 up and 83 down) between saline and IUO. When comparing the PNO and IUO groups, we found 1,465 genes (1,199 up and 266 down). We found three genes (*Sytl2*—Synaptotagmin-like 2, *Vwa5b1*—Von Willebrand Factor A Domain-Containing Protein 5B1, and a predicted gene AABR07042623.1) were differentially regulated among all three groups.

Next, the enriched biological pathways associated with these differentially expressed proteins were determined using Clue-GO analysis (Figure 3B; Supplementary Table 3). Notably, pathways involved in synaptic transmission and morphine, nicotine, and alcohol addictions were significantly enriched in the two treatment groups. Furthermore, the opioid signaling pathway was affected in the PNO and IUO offspring. A number of the genes affected in the IUO and PNO pups were associated with diseases and psychotic disorders (Table 1; Supplementary Table 4). To summarize, oxy exposure can significantly affect neurodevelopment in exposed offspring by inducing changes in key genes and pathways during synaptogenesis that could persist during late adolescence and adulthood.

**Table 1:** Differentially expressed genes identified in PFC from both IUO and PNO groups that are related to diseases or psychotic disorders.

**Disease or psychotic disorder**	**Genes associated with the disorder**
Alcohol-related birth defect	NTRK2
Alcohol withdrawal syndrome	GAD2
Anhedonia	OPRK1, PDYN
Cannabis dependence	GABRA2
Chronic schizophrenia	CYP3A4
Clinical depression	ITGAL, NPY
Cocaine dependence	OPRK1, PDYN, DRD3, GABRA2, OPRM1, NPY
Delirium	DRD3
Depression, postpartum	OPRM1
Dysphoric mood	PDYN, OPRM1
Dysthymic disorder	NTRK2
Fetal alcohol spectrum disorders	BCL2
Susceptibility to schizophrenia	MYH9, PLP1
Manic symptom	DNAH8, NR3C2, CSRP1
Non-organic psychosis	OPRM1, TBX1, CDH17, PDE10A, CSNK1E, DLX1, SP8, SP3
Paranoid schizophrenia	NTRK2
Psychoses, drug	DPYSL2, DRD1
Recurrent depression	DRD1, DRD3
Schizoaffective disorder	GRIA1, NPY, PCDH11Y, GABRB1
Schizophrenia, catatonic	CELSR1
Seasonal affective disorder	HTR2C, PER3, NPY, RORA
Severe depression	ESR2
Wernicke-Korsakoff syndrome	ALDH2

*A criterion of ±1.5-fold change, p < 0.05, and FDR < 5% was used*.

### Pain Sensitivity in Oxy-Exposed Pups

**Figure 4:**
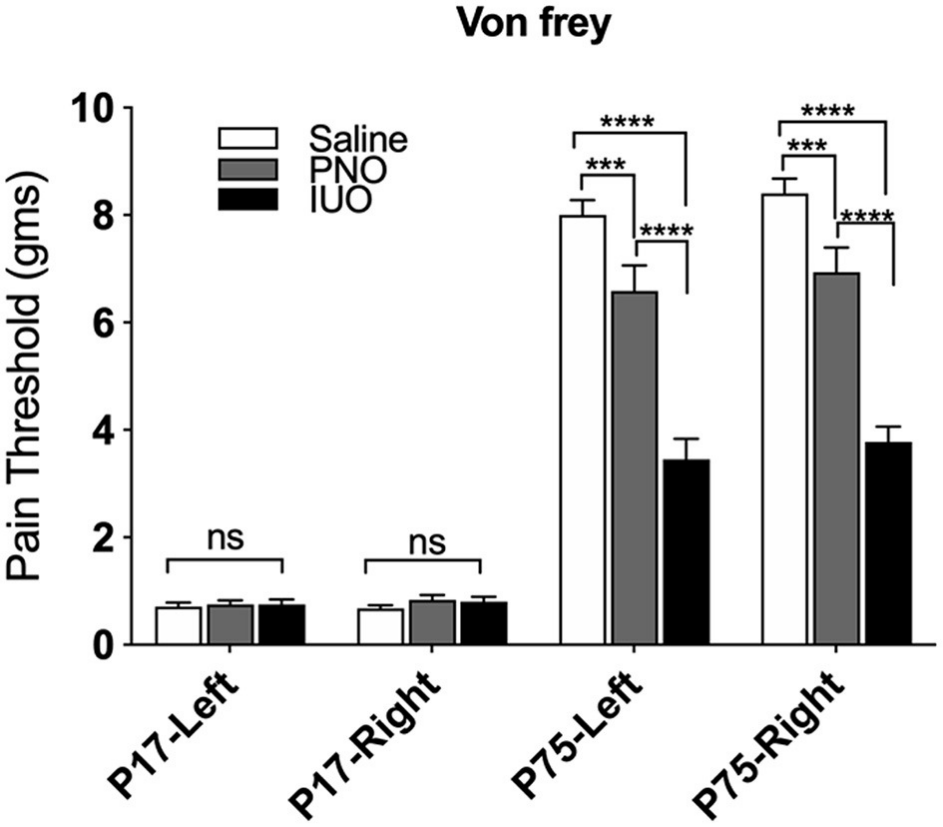
Measurement of pain thresholds using the Von Frey test. Animals from each group (*n* = 4) were tested at P17 and again at P75 to determine changes in pain thresholds. At P75, the oxy-exposed groups had lower pain thresholds than controls. Additionally, IUO pups had lower pain thresholds than PNO pups. All data represented as mean ± SEM. ***p < 0.001, ****p < 0.0001.

One key pathway we identified from our RNA-Seq analysis was regulation of sensory perception of pain. Because oxy is prescribed for pain management, we investigated whether PNO or IUO exposure has lasting effects on pain sensitivity. Von Frey testing was conducted at P17 (pups exposed to oxy via the breastmilk) and on the same animals at P75 (adulthood) after a sustained absence of oxy exposure (Figure 4). While no significant differences in the pain threshold were observed in the PNO or IUO at P17, both groups displayed a significantly lower pain threshold than controls at P75 [Age: *F*_(3, 242)_ = 455.3, *P* < 0.0001; Treatment: *F*_(2, 242)_ = 88.13, *P* < 0.0001; Interaction: *F*_(6, 242)_ = 31.18, *P* < 0.0001]. These data suggest a lasting impact of early life oxy exposure on pain sensitivity during adulthood.

## Discussion

While previous studies have reported poor neurodevelopmental outcomes in offspring exposed to opioids (Davis et al., 2010; Devarapalli et al., 2016; Sithisarn et al., 2017; Fan et al., 2018), a comprehensive analysis comparing changes in neurodevelopment with pre- and post-natal exposure to drugs has not been evaluated. In the present study we show for the first time a comparative analysis on alterations in metabolic, synaptic, molecular, and behavioral changes in offspring exposed to the oxy pre- and post-natally. As mentioned in our previous works, the PNO and IUO groups are clinically relevant (Shahjin et al., 2019; Odegaard et al., 2020). The use of both PNO and IUO groups in this study was critical to elucidate the extent of oxy exposure effects on neonates. While the PNO group was exposed to oxy only via the breastmilk, the IUO group was exposed via placental concentrations of oxycodone throughout gestation as well as via the breastmilk. Oxy as a postoperative analgesic has been reported in the literature for postpartum pain or caesarian sections in lieu of morphine drips (Niklasson et al., 2015; Nie et al., 2017). It is important to note that offspring exposed to oxy via the breastmilk may receive <10% of a typical oral therapeutic infant dose (0.1–0.2 mg/kg) (Seaton et al., 2007). Despite this low dose, infant exposure to oxy via the breastmilk has been associated with sedation and central nervous system depression (Lam et al., 2012), and a number of animal studies have also revealed deficits in behavior and development associated with perinatal opioid exposure (Davis et al., 2010; Devarapalli et al., 2016; Sithisarn et al., 2017; Fan et al., 2018).

The effects of pre- and post-natal oxy use on brain chemistry have not been clearly elucidated. Accordingly, we used ^1^H-MRS to investigate the biochemical changes present in the hippocampus of P17 rats in the PNO and IUO groups, revealing significant alterations in key brain metabolites in these groups. The IUO group had higher concentrations for the neurotransmitters ASP and GLU compared to controls and higher concentrations of NAA compared to controls and PNO. The IUO group also had lower concentrations of TAU than controls. GLU and ASP regulate a majority of the excitatory synaptic neurotransmission in the brain (Ballini et al., 2008), and their enhanced expression may point to excitotoxicity and possibly enhanced excitatory signaling in the brain. Additionally, higher concentrations of ASP may suggest more ASP is available to react with acetyl-CoA to make NAA (Hajek and Dezortova, 2008), which was also elevated in the IUO group. Interestingly, TAU plays a key role in brain development, and its deficiency can lead to a delay in cell differentiation and migration in certain brain areas such as cerebellum, pyramidal cells, and visual cortex (Ripps and Shen, 2012). Further, Hernandez-Benitez et al. have shown that TAU promotes neural development in the embryonic brain as well as in adult brain regions (Hernández-Benítez et al., 2010). The lower levels of TAU we observed may point to neurodevelopmental deficits. Intriguingly, we have shown that both IUO and PNO animals display an overall reduction in the head size circumference (Odegaard et al., 2020), which may be attributed in part to lower concentration of TAU.

Based on the observation of increased GLU levels in our MRS study, we investigated the extent of synaptic changes in IUO and PNO animals. Glutamate receptors play a role in mediating the reward pathway involved in drug addiction (D'Souza, 2015), and they are also involved in opiate-induced neural and behavioral plasticity (Jackson et al., 2000; Trujillo, 2000; Zhu and Barr, 2004). AMPA receptors, one type of ionotropic glutamate transporter, are crucial for opioid withdrawal during development (Jakowec et al., 1995a,b; Fitzgerald et al., 1996; Washburn et al., 1997; Mahanty and Sah, 1998; Ozawa et al., 1998). While we saw a reduction in the AMPA/NMDA ratio in the PNO rats, the difference was not significant. Additionally, there were no differences in the PPR of post-synaptic currents, suggesting no changes in vesicle release. Intriguingly, PNO mEPSCs had slightly faster decay kinetics, which is consistent with altered AMPA receptor subunit composition. Interestingly, no significant effects were seen in the IUO group. Possible reasons include the potential loss of neurons given the longer exposure to oxy (Hu et al., 2002; Hauser and Knapp, 2017) and the higher glutamate levels in the IUO pups compared to the PNO group, as evidenced by ^1^H-MRS. Thus, our study for the first time lends insight into the synaptic changes associated with PNO exposure and its effects on altered glutamatergic signaling.

Recent studies employing high-throughput technologies have further provided new inroads in elucidating the molecular underpinnings associated with long term oxy dependency. These include alterations in key genes associated with integrated stress response in the brain (Fan et al., 2015), induction of apoptotic signaling in neurons by promoting demyelination (Fan et al., 2018), alterations in reward related genes (Zhang et al., 2018), axon guidance molecules (Yuferov et al., 2018), inflammation/immune-related genes (Zhang et al., 2017), neurotransmitter receptor genes (Zhang et al., 2014), and synaptic plasticity genes (Zhang et al., 2015), including key sex-specific neuroplasticity-related genes (Randesi et al., 2018). Similarly, our RNA-seq data showed alterations in pathways associated with synaptic transmission, axon guidance, inflammasomes, and genes associated with the reward system. Among others, genes affecting synaptic transmission and axon development included *Egfr, Adrb2*, and *Ntrk*. Interestingly, Fan et al. found that chronic oxy exposure leads to axonal degeneration in rat brains (Fan et al., 2018). Chronic oxy exposure altered the white matter of the rats via deformation of axonal tracks, reduced size of axonal fascicles, loss of myelin basic protein, and accumulation of the amyloid precursor protein (Fan et al., 2018). Importantly, human studies of infants prenatally-exposed to opioids have shown alterations in the white matter, such as punctate white matter lesions or white matter signal abnormalities on structural MR imaging (Walhovd et al., 2012; Merhar et al., 2019). The results from our RNA-seq analysis align with these previous observations from both animal models and human studies. Interestingly, our RNA-seq data showed differences in glutamatergic synapse genes, with seven genes being differentially expressed in the IUO and PNO groups: *Adrb2, Egfr, Grik2, Npy2r, Ntrk1, Ntrk2*, and *Oxtr*. Combined with our electrophysiology results, our RNA-seq results further suggest alterations in glutamatergic signaling within the reward pathway of these exposed offspring. In addition, our studies suggest PNO and IUO exposure not only alter gene expression but also may increase the risk of developing other diseases, particularly renal disease. Genes associated with renal adysplasia, renal cancers, renal failure, and several other renal diseases were differentially regulated in the PNO and IUO groups. Interestingly, depletion of TAU, such as that reported in our IUO group MRS data, has been shown to play a role in renal dysfunction (Ripps and Shen, 2012). In human studies, opioid use has been associated with acute kidney injury, particularly in the case of opioid overdose (Mallappallil et al., 2017). The altered gene expression and lower levels of TAU in the reward system of the IUO offspring during early development may warrant further exploration into the potential of these offspring to develop renal diseases as adults. Further, several genes in our analysis are also involved in other substance use-related disorders, such as nicotine, cocaine, and cannabis dependence, morphine addiction, and fetal alcohol spectrum disorders. Opioid use has also been associated with mental health disorders, with a higher proportion of adolescents exposed prenatally to opioids having experiences with major depressive episodes, alcohol abuse, and attention deficit hyperactivity disorder (Nygaard et al., 2019). Genes associated with depression, anxiety disorders, schizophrenia, and obsessive compulsive disorder were all enriched in both PNO and IUO offspring, suggesting a higher risk for such disorders in these offspring.

Oxy is generally prescribed for pain management, and the enrichment of the sensory pain pathway from our RNA-Seq analysis was not surprising. When assessed by the Von Frey filament test at P17, the PNO and IUO pups did not exhibit a difference in pain threshold compared to saline controls. However, during adulthood (P75), these same animals, especially the IUO group, displayed significantly lower pain threshold compared to saline offspring. In a study of neonatal morphine exposure, P40 rats exhibited a lower pain threshold than the controls, but the pain threshold approached control levels by P50 when tested using Von Frey (Zhang and Sweitzer, 2008). Because we see similar results continuing up to P75, pre- and post-natal exposure to oxy may alter normal synaptic development involved in nociception. Indeed, a number of genes shown to be differentially regulated in both PNO and IUO in our RNA-seq data are associated with pain: *Adrb2, Cck, Htr2c, Npy2r, Oprk1, Oprm1*, and *P2rx3*. Further analyses of these genes could possibly lend more mechanistic insights into the pain etiology in these offspring.

In summary, our study using a holistic systems approach shows a comparative analysis on alterations in metabolic, synaptic, molecular, and behavioral changes in offspring exposed to the prescription opioid oxy pre- and post-natally. Importantly, these changes not only impact the overall development during early stages but also persist into adulthood.

## Data Availability Statement

The datasets presented in this study can be found in online repositories. The names of the repository/repositories and accession number(s) can be found at: https://www.ncbi.nlm.nih.gov/geo/, GSE159563.

## Ethics Statement

The animal study was reviewed and approved by Institutional Animal Care and Use Committee of the University of Nebraska Medical Center (UNMC).

## Author Contributions

KO: animal treatments and maintenance, interpreted results, and drafted and edited manuscript. VS: animal treatments and maintenance, project organization, and scheduling. AC and SK: animal treatments and maintenance. JS: bioinformatic analysis of RNA-seq data and data deposition. ZX and HW: Von Frey behavior testing, analysis, and figures. MM, YL, and MU: MRI/MRS acquisition and analysis. AS and MV: electrophysiology experiments, analysis, and figures. CG: bioinformatic analysis of RNA-seq data. SL: conception of the experimental design and provision of resources. GP and SY: conception of the experimental design, oversaw experiments and analyses, and edited manuscript. All authors contributed to the article and approved the submitted version.

## Conflict of Interest

The authors declare that the research was conducted in the absence of any commercial or financial relationships that could be construed as a potential conflict of interest.
